# Association of Retinal Biomarkers, Sleep Quality, and Daytime Sleepiness with Cognitive Impairment in the Elderly Population in Taiwan

**DOI:** 10.1002/alz70856_098837

**Published:** 2025-12-24

**Authors:** Ting‐Wen Chu, Yi‐Ting Hsieh, Jen‐Ming Chiou, Yao‐Lin Liu, Jen‐Han Chen, Yen‐Ching Chen

**Affiliations:** ^1^ Institute of Epidemiology and Preventive Medicine, College of Public Health, National Taiwan University, Taipei, Taiwan; ^2^ Department of Ophthalmology, Mackay Memorial Hospital, Taipei, Taiwan; ^3^ Department of Medicine, MacKay Medical College, New Taipei City, Taiwan; ^4^ National Taiwan University Hospital, Taipei, Taiwan; ^5^ Institute of Statistics and Data Science, National Taiwan University, Taipei, Taiwan; ^6^ Institute of Statistical Science, Academia Sinica, Taipei, Taiwan; ^7^ Department of Ophthalmology, Far Eastern Memorial Hospital, New Taipei City, Taiwan; ^8^ Department of Geriatrics and Gerontology, National Taiwan University Hospital Yunlin Branch, Yunlin, Taiwan; ^9^ Department of Medicine, College of Medicine, National Taiwan University, Taipei, Taiwan; ^10^ Department of Public Health, College of Public Health, National Taiwan University, Taipei, Taiwan

## Abstract

**Background:**

The early detection of preclinical dementia is critical, prompting research into retinal biomarkers using optical coherence tomography (OCT). Limited longitudinal studies have investigated the association between retinal biomarkers‐ retinal nerve fiber layer (RNFL) and ganglion cell‐inner plexiform layer (GC‐IPL) thickness, and sleep pattern, including sleep quality and daytime sleepiness, with cognitive function over time. This study aims to explore these relationships in non‐demented older adults.

**Method:**

This four‐year prospective cohort study (2015‐2022) involved 257 non‐demented older adults at baseline (2015‐2017) from the ongoing Taiwan Initiative for Geriatric Epidemiological Research. Cognitive function was assessed at baseline and two follow‐ups through global and domain‐specific measures, including memory, attention, executive function, and verbal fluency, using the Montreal Cognitive Assessment–Taiwanese version (MoCA‐T) and a battery of neuropsychological tests, respectively. Retinal data were collected using OCT, and sleep quality and daytime sleepiness were assessed via the Pittsburgh Sleep Quality Index (PSQI) and the Epworth Sleepiness Scale (ESS) at baseline. PSQI scores >5 indicate poor sleep quality, while ESS scores ≥11 suggest a high risk of excessive daytime sleepiness. Multilevel models were utilized to examine the associations of RNFL and GC‐IPL thickness and sleep pattern with cognitive function, adjusting for covariates.

**Result:**

Increased baseline RNFL and GC‐IPL thickness were associated with poorer global cognition at baseline (MoCA‐T: β=‐0.73×10^−2^ and β=‐1.12×10^−2^, respectively) but exhibited protective effects on global cognition over time (MoCA‐T: β=0.39×10^−2^ and β=0.60×10^−2^, respectively). Baseline GC‐IPL thickness was associated with poorer attention at baseline (digit span backward test: β= ‐0.28 × 10^‐2^), but demonstrated protective effects on the performance of memory and attention over time (logical memory: β= 0.06 × 10^‐2^, digit span backward: β= 0.15 × 10^‐2^). Stratified analyses revealed significant interactions between the status of sleep quality and GC‐IPL thickness for global cognition and attention (*P_interaction_* <0.01). Similarly, stratified analyses revealed significant interactions between the status of excessive daytime sleepiness and GC‐IPL thickness for global cognition and memory (*P_interaction_* <0.01).

**Conclusion:**

Retinal biomarkers exhibit non‐linear associations with cognitive function, driven by an interactive relationship between sleep quality and daytime sleepiness. These findings suggest that retinal and sleep pattern assessments may enhance predictions of cognitive health.

